# Optical coherence tomography to identify upper airway obstruction sites in an apneic patient

**DOI:** 10.1117/1.BIOS.1.3.035002

**Published:** 2024-11-28

**Authors:** Joseph C. Jing, Khodayar Goshtasbi, Yong Wang, Jason J. Chen, Erica Su, Ellen M. Hong, Katelyn D. Dilley, Yan Li, Frances B. Lazarow, Anthony Chin Loy, David Shamouelian, Said E. Elghobashi, Zhongping Chen, Brian J. F. Wong

**Affiliations:** a University of California Irvine, Beckman Laser Institute, Irvine, California, United States; b University of California Irvine, School of Biomedical Engineering, Irvine, California, United States; c University of California Irvine, Department of Otolaryngology-Head and Neck Surgery, Irvine, California, United States; d University of California Irvine, Department of Mechanical and Aerospace Engineering, Irvine, California, United States

**Keywords:** optical coherence tomography, obstructive sleep apnea, upper airway imaging, sleep surgery, sleep endoscopy, airway 3D reconstruction

## Abstract

**Significance:**

There are important limitations to the current diagnostic tools for obstructive sleep apnea (OSA).

**Aim:**

The limitations warrant the development of a more accurate, minimally invasive, and objective tool to better characterize upper airway airflow obstruction during sleep.

**Approach:**

Swept-source optical coherence tomography (OCT) as a range-finding technique was utilized for upper airway volumetric reconstruction. An acousto-optic modulator was integrated into the OCT system for extending the imaging range to be suitable for the typical diameter of the upper airway. High spatial resolution images were acquired at 25  frames/s and translated at 12.5  mm/s before manual segmentation for three-dimensional (3D) reconstruction and numerical analysis. The Lattice-Boltzmann method as a computational fluid dynamics (CFD) technique was utilized to pinpoint the precise locations of turbulent airflow in the airspace.

**Results:**

Upper airway OCT imaging of a 28-year-old individual with sleep disorder breathing was obtained during awake and sleep periods. The volumetric structure and 3D reconstruction of the upper airway through a micrometer-resolution optical imaging approach were successfully demonstrated. Cross-sectional volumetric changes from awake to sleep periods were calculated, and the greatest airway obstruction was observed at the level of the oropharynx. In addition to CFD analysis, measuring airflow pressure differences along the upper airway aided in accurately localizing the dominant sites of obstruction during sleep.

**Conclusion:**

The combination of the proposed OCT imaging system with 3D remodeling and CFD analysis led to accurate reconstruction of the upper airway and identification of obstruction sites during sleep. This technology can improve surgical decision-making and outcomes in OSA.

Statement of Discovery We describe an OCT imaging system with 3D modeling for accurate reconstruction of upper airway and identification of site of obstruction during sleep apnea.

## 1 Introduction

Obstructive sleep apnea (OSA) affects 9% to 38% of the adult population,^1^ and the corresponding economic impact can be up to $150 billion annually.^2^ If inadequately diagnosed or treated, OSA can lead to severe health complications such as cardiovascular events, neurocognitive effects, and depression.^3^ The gold standard diagnostic tool for OSA is polysomnograms, which measure important parameters such as the apnea–hypopnea index, percutaneous oxygen content, rapid eye movement sleep, and limb movements.^4^ However, polysomnograms cannot provide information regarding the structure and anatomical collapse of the airway. The upper airway is a complex anatomical structure that undergoes dynamic geometric changes over the respiratory cycle. These structural changes can cause airway compromise at many locations, including the palate, base of the tongue, pharyngeal wall, epiglottis, and larynx. The accurate identification of the sites of airway obstruction during sleep is essential for determining the ideal medical or surgical management. Drug-induced sleep endoscopy (DISE)^5^ is an emerging method to identify the obstruction sites during sleep and is now widely implemented in surgical practice. However, the interpretation of DISE can be subjective with interobserver disagreement in the location of airway obstruction.^6^ Despite best efforts utilizing multiple diagnostic approaches, a large number of sleep patients (55% to 57%)^7^ still fail to adequately respond to surgical interventions. As such, the fields of sleep medicine and sleep surgery are in need of better diagnostic tools for the evaluation of upper airway collapse during sleep.

Optical coherence tomography (OCT) is a well-established mesoscopic imaging modality that can obtain non-invasive, high-speed, and high-resolution images of biological systems.^8^ OCT can couple into a sectional miniature imaging catheter and be used as a range-finding modality to determine the size and shape of upper airway anatomical structures.^9^^–^^15^ In a 2012 study by Jing et al.,^16^ a long-range OCT system was used to rapidly scan the upper airways of three awake human subjects. This system generated a volumetric dataset that reproduced the airway lumen with an accuracy within the order of tens of microns. The present study aims to utilize a similar OCT setup to acquire high-resolution three-dimensional (3D) reconstructions of the upper airway lumen in an apneic patient during sleep. By accounting for the curvature of the dynamic airway in the volumetric reconstruction, we theorize that the respiratory airflow modeling will provide a rigorous method for identifying sites of obstruction in OSA.

## 2 Methods

### 2.1 Long-Range OCT System

**Fig. 1:**
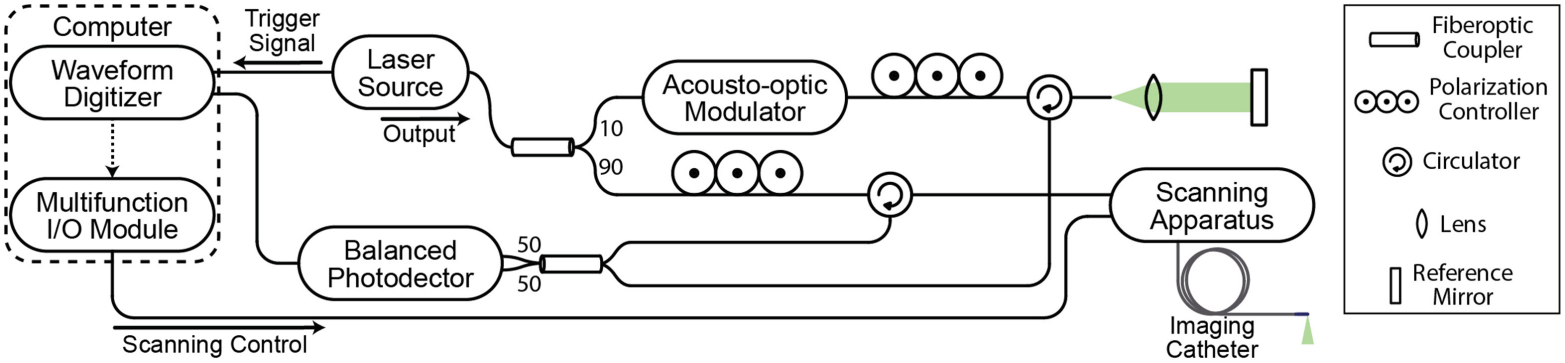
Schematic of the OCT system for upper airway imaging.

**Fig. 2:**
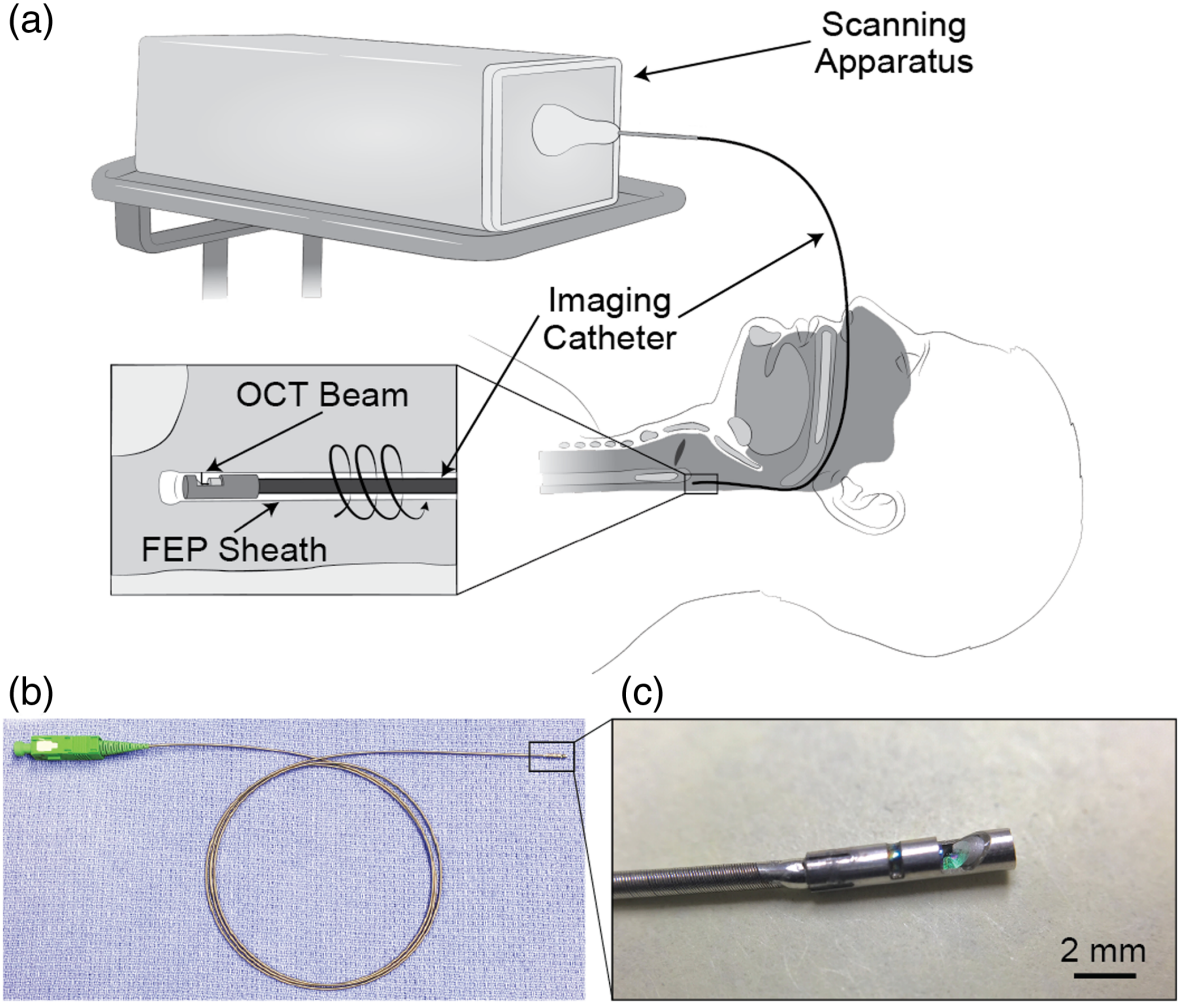
Upper airway OCT catheter. (a) The schematic of the imaging procedure for upper airway imaging. The OCT catheter is housed in a transparent FEP sheath that is inserted through the nasal cavity and positioned in the hypopharynx. The 90-deg imaging catheter rotates and retracts within the FEP sheath during data acquisition. (b) Photograph of the OCT catheter. (c) Close-up photograph of the catheter tip.

As depicted in Fig. 1, the OCT system utilized in this study incorporates an acousto-optic modulator (Brimrose Corp. of America, Sparks Glencoe, Massachusetts, United States) to generate a frequency shift of 150 MHz in the interferometer signal. A full imaging range of 12.8 mm at 6 dB was achieved by removing signal processing artifacts.^17^ The swept-source laser (Axsun Technologies, Billerica, Massachusetts, United States) had a center wavelength of 1310 nm with a bandwidth of 109 nm, allowing for an axial resolution of 10  μm in tissue (assuming a refractive index of 1.4). The fiberoptic-based imaging catheter consisted of a gradient-index rod lens for focusing and a 45-deg mirror at its distal tip to achieve an endoscopic 90-deg-angle view (Fig. 2). The optical assembly at the tip was protected within a stainless-steel housing with a lateral opening for imaging beam propagation. The metal housing had an outer diameter of 1.5 mm and was firmly soldered to a stainless-steel torque coil, which contained the single-mode optical fiber that connected the light source to the imaging optics. The torque coil had an outer diameter of 0.97 mm. Driven by a brushless direct current (DC) motor at the proximal end, the entire catheter was rotated through the torque coil during imaging acquisition. Each complete rotation of the catheter provided a two-dimensional cross-sectional image, or a B-scan. Volumetric imaging was achieved by pulling back the catheter using a motorized translation stage during rotation to acquire a series of B-scans with equal spacing. The DC motor and the translational stage together comprised the scanning apparatus. Imaging was performed at a rotational rate of 25  frames/s with 2000 axial scans per image and a pullback rate of 12.5  mm/s.

### 2.2 Image Acquisition

During imaging, the entire catheter rotated within a sealed fluorinated ethylene propylene (FEP) sheath, which ensured probe sterility as well as the safety of the patient. The patient was locally anesthetized and decongested with lidocaine HCl 4% and phenylephrine HCl 1% spray. The transparent FEP sheath containing the imaging catheter was inserted into the nose and guided through the nasopharynx and oropharynx to the pyriform sinus. The patient was asked not to speak or swallow during imaging. The rotation and translation of the imaging catheter were initiated, whereas the sheath remained stationary. Leaving the sheath in place allowed for multiple imaging passes without re-inserting the sheath-catheter apparatus. Each imaging run was 30 s and spanned a distance of ∼15 to 20 cm (from pyriform sinus to nare). The total number of images depended on the airway length, depth of catheter insertion into the airway, and catheter pullback speed. If the initial pullback indicated that the catheter was not inserted deep enough into the airway, the catheter was adjusted prior to the next imaging run. To characterize the natural anatomical curvature of the airway for reconstruction, a 3D electromagnetic tracking system (Ascension Technology Corp., Shelburne, Vermont, United States) was inserted through the sheath after the imaging catheter was removed to obtain the spatial coordinates of the catheter passage. Imaging was performed supine while the patient was awake and then repeated, with the sheath remaining in place, after the patient spontaneously fell asleep, which was confirmed by snoring. The sheath was taped to the patient’s lip/cheek to avoid movement, and the patient was held still as much as possible to prevent any sheath mobility or instabilization. All imaging procedures were reviewed and approved by the Institutional Review Boards at the University of California, Irvine prior to all experiments.

### 2.3 Image Processing and 3D Reconstruction

Image sets were assessed for overall quality prior to 3D reconstruction. Image quality was evaluated by considering signal intensity, distortion caused by non-uniform rotation of the imaging catheter, presence of artifacts/noise, identifiable anatomical structures, and limitations of imaging range. A detailed description of the data selection process has been previously reported in a 2015 study by Lazarow et al.^18^ Only image sets that demonstrated high signal-to-noise ratio, minimal to no distortion, and identifiable anatomical structures were rendered into 3D models.

**Fig. 3:**
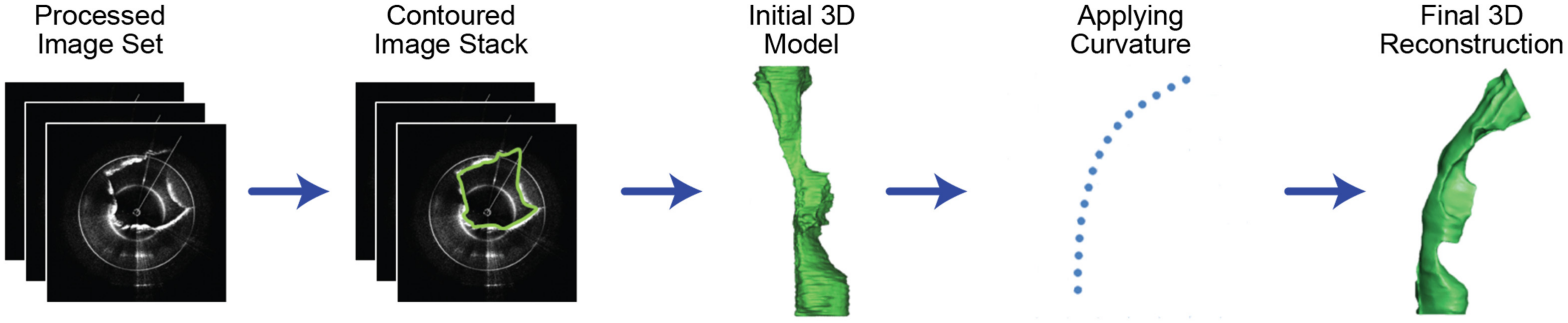
Flowchart describing the process of 3D reconstruction from OCT images.

Raw images were processed and analyzed using IrfanView (IrfanView, Irfan Skiljan, Austria), MATLAB (MathWorks, Natick, Massachusetts, United States), and Amira (FEI Visualization Science Group, Burlington, Massachusetts, United States). The technique used to create 3D reconstructions of the upper airway from OCT images has been detailed in a 2014 study by Nguyen et al.^19^ Dispersion correction was not further performed in this system. The images were post-processed for correct anatomical scaling, sequential image alignment, image downscaling, and converting from Cartesian to polar coordinates. The processed images were imported into Mimics (Materialise, Leuven, Belgium) for manual segmentation to define the contour of the airway lumen at each B-scan. The B-scans of the contour were assembled into a “cylindrical” representation of the airway. To recreate the anatomical curvature of the native airway, the spatial coordinates of each B-scan were adjusted based on the curvature obtained using the 3D electromagnetic tracker using a custom morphing algorithm in Visual Basic (Microsoft, Redmond, Washington, United States). If important anatomical structures (e.g., tracheal wall) were outside or at the periphery of the imaging range, often the pre- and post-frame images could be used to manually extrapolate the structures. The anatomically accurate 3D model was created in Mimics, and the excess proximal and distal ends of the model were trimmed off using Netfabb Basic (Netfabb, Parsberg, Germany) (Fig. 3).

### 2.4 Computational Fluid Dynamics

The airflow in the complex geometry of the upper airway undergoes a transition from laminar to turbulent and reverses its direction within the respiratory cycle.^20^ Direct numerical simulation (DNS) as a computational fluid dynamics (CFD) approach resolves the relevant spatial and temporal scales and thus produces accurate details of turbulent flow properties. Specifically, the Lattice Boltzmann method (LBM) method was employed for solving discretized lattice Boltzmann equations^21^^,^^22^ because DNS-LBM was well-suited for resolving the relevant length and time scales of flows in the complex geometry of the upper airway. In a 2014 study, Wang and Elghobashi^23^ developed a DNS-LBM method, which predicted airflow obstruction sites based on 3D reconstruction models of CT scans. Here, the same DNS-LBM solver and its parallel computing were utilized to locate the sites of turbulent flow in the upper airway. Both single-relaxation time (SRT)^24^ and multi-relaxation time (MRT)^25^ collision operators were considered in the DNS-LBM solver. The MRT operator allowed the solution to be more stable than that of SRT at the cost of more computational time. More detailed descriptions of the DNS-LBM approach are presented in the Supplementary Material. The respiratory cycle of the patient was separately measured as ∼5  s, and this timeframe was considered when performing the CFD analysis of images within a hypothetical respiratory cycle.

## 3 Results

**Fig. 4:**
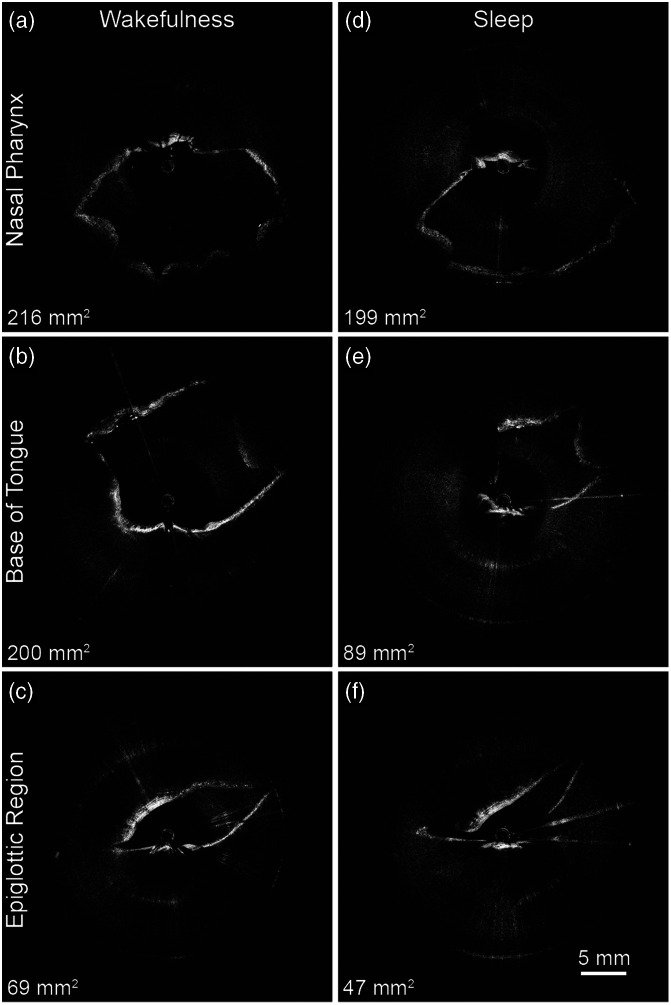
Upper airway OCT images of the studied subject (sleepiness score of nine and a BMI of 27·6). (a)–(c) Images taken during wakefulness, and (d)–(f) images taken during wakefulness. The corresponding cross-sectional area measurements are noted (Video 1, MP4, 4.25 MB [URL: https://doi.org/10.1117/1.BIOS.1.3.035002.s1]).

A 28-year-old male patient with a body mass index of 27.6 who reported nighttime snoring and had an Epworth Sleepiness score of nine was enrolled in the study. Figure 4 represents the awake (4a-c) and asleep (4d-f) results of the upper airway reconstruction. As the most intuitive parameter in describing airflow, the measurements of airspace cross-sectional area at each B-scan were calculated using ImageJ,^26^ and the results for the nasopharynx [Figs. 4(a) and 4(d)], base of tongue [Figs. 4(b) and 4(e)], and epiglottic region [Figs. 4(c) and 4(f)] are shown in each subfigure. More than 85% of the image volume data were analyzable with a high degree of measurement confidence. The cross-sectional areas are reduced by 8%, 56%, and 32% in the nasopharynx, base of the tongue, and epiglottic regions, respectively.

**Fig. 5:**
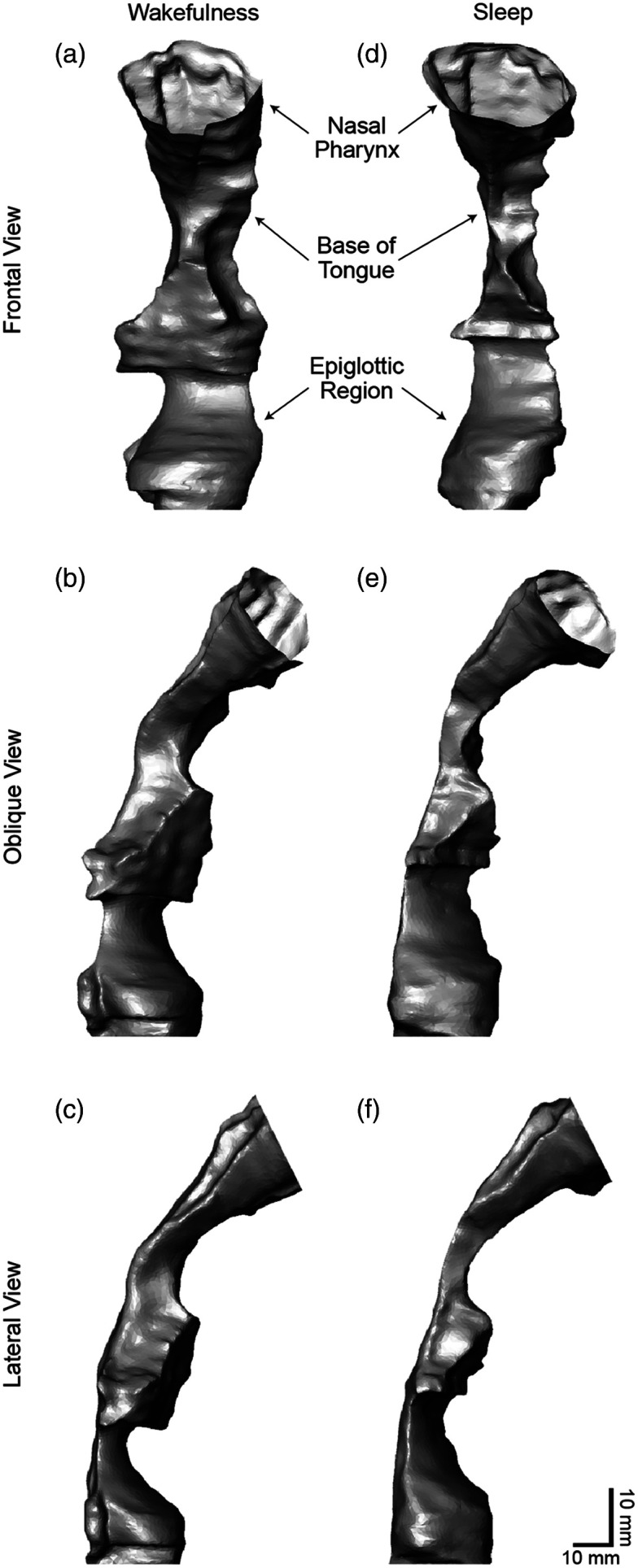
Frontal, oblique, and lateral views of volumetric reconstruction of the upper airway lumen space. (a)–(c) Data acquired during wakefulness and (d)–(f) during native sleep (Video 2, MP4, 16.32 MB [URL: https://doi.org/10.1117/1.BIOS.1.3.035002.s2]).

During the experiment, OCT was displayed in real time during the rotation and retraction of the imaging catheter. This is demonstrated by Video 1, which shows the OCT video streams during wakefulness and sleep. The 3D reconstructions of the corresponding airway lumens are presented in Fig. 5 in the frontal, oblique, and lateral view, redemonstrating the severe degree of oropharyngeal obstruction during sleep. Video 2 presents a paired virtual fly-through of the airway models reconstructed from Fig. 4 to graphically illustrate the differences between wakefulness and sleep.

**Fig. 6:**
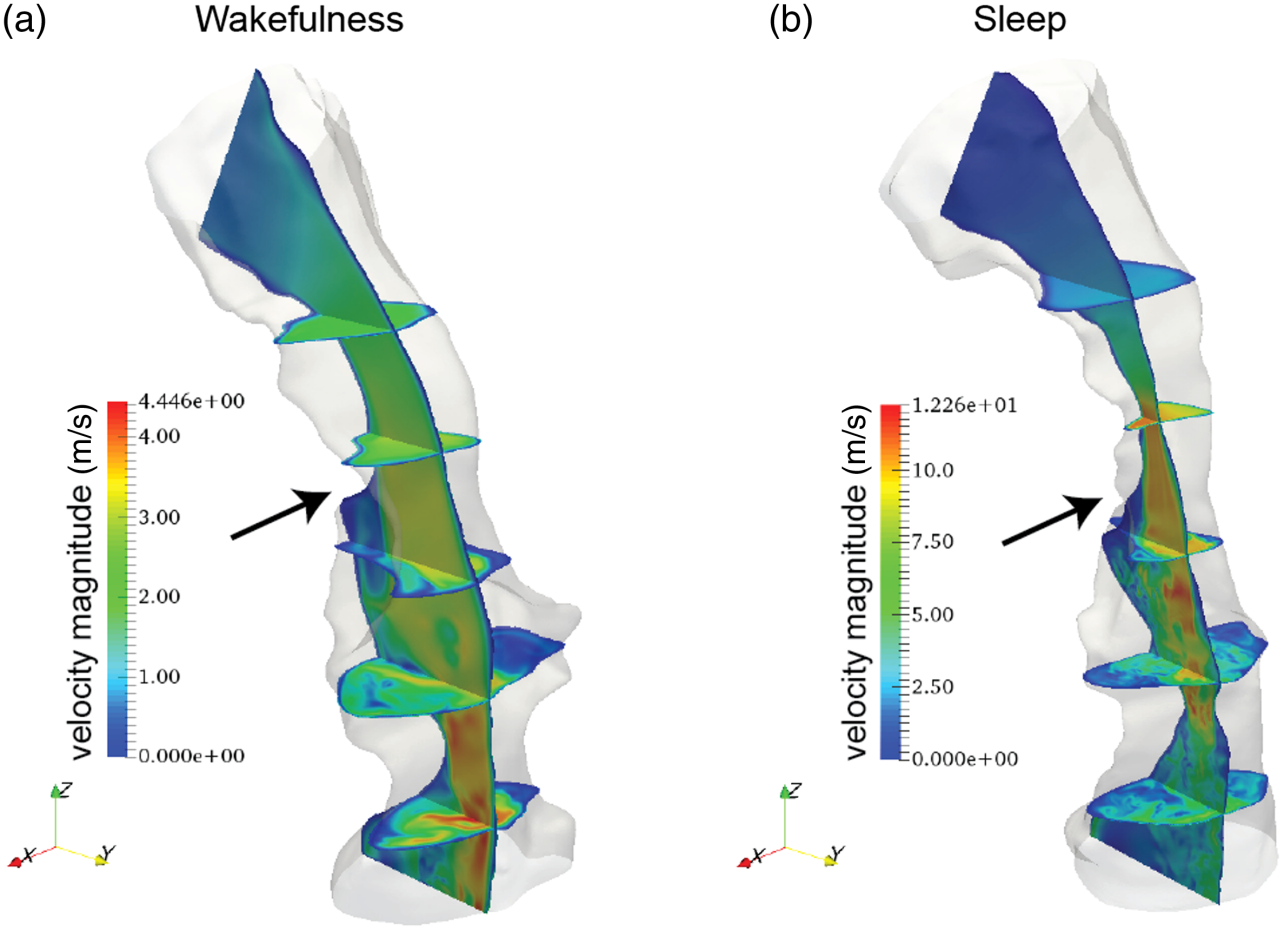
Velocity contours from simulations based on a flow rate of 9.45  L/min. (a) Simulation based on wakefulness data. (b) Simulation based on native sleep data. Arrows indicate the most severe sites of obstruction.

CFD analysis of airflow was used to understand the physics of respiration in wakefulness and sleep. Currently, practical approaches for direct measurement of instantaneous velocity and pressure within the complex geometry of the human upper airway are lacking, and therefore, numerical simulations are of great value. DNS-LBM was used to calculate changes in velocity and pressure in fluid flow. The resulting velocity magnitude contours from the DNS-LBM are illustrated in Fig. 6. They demonstrate the acceleration of the airflow through anatomical regions with reduced cross-sectional area, where a high-velocity flow was most notable at the base of the tongue region indicated by the arrows in Fig. 6. As the tongue retruded, the flow velocity increased profoundly at the base of tongue of this patient.

**Fig. 7:**
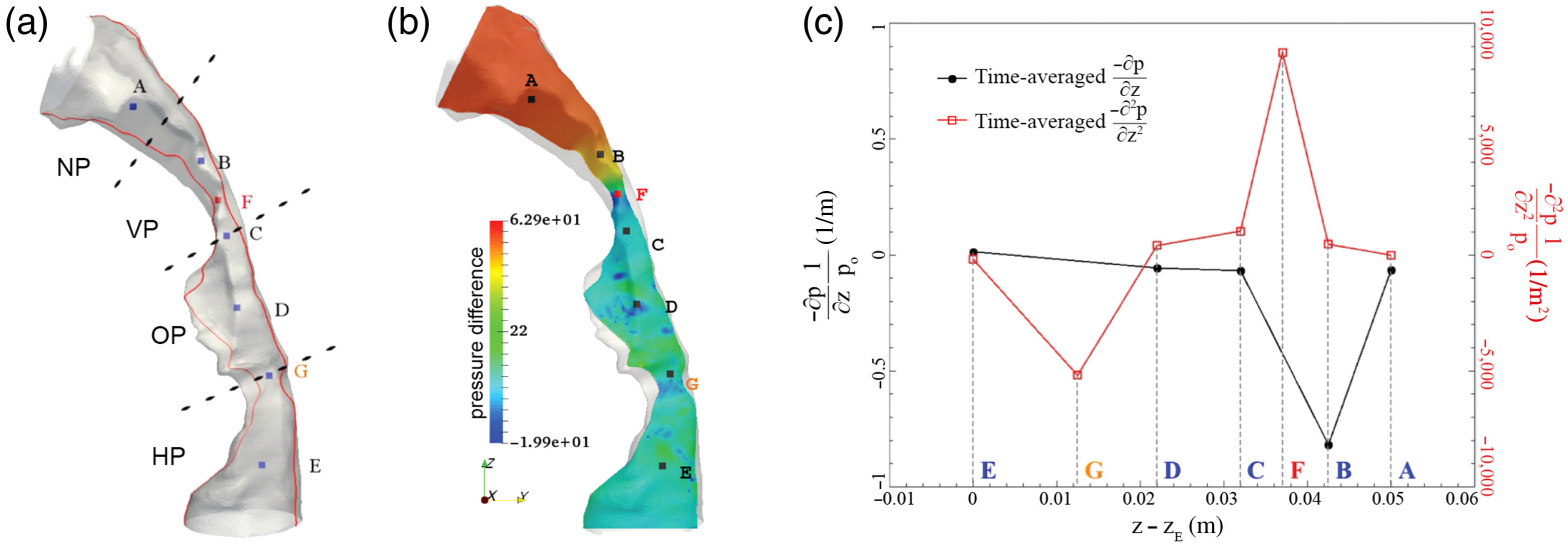
In accordance with previously published methods,^23^ an upper airway model indicating the locations at which time-averaged ∂p/∂z and ∂2p/∂z2 were calculated. (a), (b) Pressure differences within the airway model. (c) Calculated time-averaged ∂p/∂z and ∂2p/∂z2 at each location. (NP, nasopharynx; VP, velopharynx; OP, oropharynx; HP, hypopharynx).

Figure 7(a) is a structural depiction of the patient’s airway with seven anatomical points of interest and corresponding airflow obstruction levels, an approach that was previously validated by Wang and Elghobashi.^23^ The pressure differences shown in the false color scale in Fig. 7(b) demonstrate a local minimum at point F, namely, at the patient’s base of the tongue. The calculation of the time-averaged second spatial derivative of the pressure difference $\partial^{2}p/\partial z^{2}$ could accurately pinpoint the major obstruction sites, as shown in Fig. 7(c).

## 4 Discussion

Accurately targeting sites of airway obstruction leads to improved surgical decision-making and outcomes in OSA patients. In this study, high-fidelity structural information and airflow data of a sleeping patient’s upper airway were obtained through OCT imaging and 3D reconstruction, allowing for precise identification of various anatomical sites with airflow obstruction. The resultant models demonstrate a severe degree of oropharyngeal obstruction during sleep by depicting the locations of significant airway lumen collapse as determined by cross-sectional measurements. Although information on the site of airway occlusion during sleep may be inferred from the structural images alone, a more comprehensive understanding of its physics is gained from CFD analysis of the airflow. With the 3D reconstruction from the OCT scans, we performed airflow simulations through a sophisticated algorithm to calculate pressure and velocity profiles. By examining local pressure differences, the dominant site of airway obstruction could be accurately identified. This is contrary to current diagnostic tools such as polysomnograms and DISE, with major limitations lending to their subjective nature. The proposed OCT upper airway imaging through a fiberoptic catheter approach combined with CFD analysis is an improved diagnostic and surgical decision-making tool for sleep surgeons and may increase the treatment success rate for OSA patients.

The reported methodology for OCT catheter imaging is minimally invasive and can be performed during native sleep without the need for anesthetics or an operating room. This provides a novel, accurate, and low-cost pathway to quantitatively assess upper airway geometry and obstruction of patients suffering from OSA. Unlike polysomnography, OCT directly visualizes the upper airway lumen geometry, which can be critical in identifying the anatomical changes that occur during sleep. OCT imaging is also superior to other imaging modalities that have been historically utilized in OSA evaluation such as computed tomography (CT) or magnetic resonance imaging (MRI).^27^^,^^28^ Ultrafast (Cine) CT and MRI are available, but Cine CT exposes patients to significant ionizing radiation, whereas Cine MRI is loud (disrupting sleep) and lacks adequate temporal and spatial resolution for comprehensive dynamic imaging. The instrumentation and imaging catheters utilized in this study are physically similar to commercial *in vivo* pH monitors and nasogastric feeding, making them low-profile and unobtrusive. The imaging catheter operates within a protective sheath that has a 1.8-mm diameter, but it can be miniaturized to further reduce discomfort, improving the ease of imaging especially during native sleep. Upper airway OCT catheters with a diameter of <1  mm have been previously reported.^29^^,^^30^ As the diameter of the airway lumen is typically up to 25 mm, the imaging optics should possess a focal length that is approximately similar to that. Typical focusing optical components such as convex lenses or gradient-index rod lenses are the limiting factors for the miniaturization of the imaging catheter. Alternatively, a graded-index fiber can be fused with the transmitting single-mode optical fiber to reduce the catheter diameter. All in all, the minimally invasive, highly precise, and low-cost nature of OCT positions it as a superior imaging modality to characterize OSA in the future.

Although this study focuses on the utilization of OCT as a range-finding device to acquire airway lumen volume, it is worth noting that OCT can provide cross-sectional tissue information and can be used to study morphological abnormality within the upper airway at the cellular level.^31^ OCT systems powered by longer wavelength swept-source lasers have been demonstrated for enhanced imaging depth^32^^,^^33^ and may be adapted in upper airway imaging where abundant cartilage tissue exists. Furthermore, OCT imaging catheters can be integrated with supplementary modalities, such as near-infrared fluorescence imaging that identifies inflammatory activity at the molecular level,^34^^–^^36^ which can provide additional information regarding the disease pathology of OSA. OCT-based elastography techniques have also been proposed to study tissue mechanical response in the upper airway.^37^^–^^39^ These techniques can bring further insights into the mechanisms of OSA at the cellular level by studying *in vivo* tissue morphological and mechanical changes.

This proposed methodology for characterizing OSA with OCT has some limitations. First, this study had only one subject, and there were no healthy controls. As such, future studies with multiple OSA and healthy subjects for thorough comparisons are warranted. Next, high-speed swept-source lasers have become commercially available and can provide an approximately eightfold improvement in imaging speed while maintaining the imaging range when adapted into our current imaging system. However, the normal respiration rate is about seven breaths per minute,^40^ or ∼8.6  s per breath, and our current OCT setup takes ∼20  s to complete each pullback (with the spacing of 500  μm between B-scans). Therefore, we cannot finish data acquisition within a respiratory cycle. A higher imaging speed that enables faster pullback would be especially advantageous because the upper airway geometry varies over the course of the respiratory cycle. Quicker airway lumen imaging decreases the opportunity for geometric change, thus increasing the accuracy of CFD analysis. Another limitation is that manual extrapolation was done for several images where the airway would fall in proximity or outside of the imaging range, which could affect the accuracy of the results, and as such future studies should have extended imaging range to combat this limitation. Furthermore, the OCT raw images have a variable degree of artifacts (as is also shown in Fig. 4), which can theoretically become indistinctive from airway walls or other important structures on occasion. These artifacts can arise from photodetector saturation due to a large degree of specular reflection off the protective sheath. In this paper, user-defined spatial filters delineated artifacts away from the airway wall when they were in contact or indistinctive, and thus, future studies need to make this process both more accurate and expedited. In the future, the artifact phenomenon can also be reduced by several methods such as subtracting superficial plexuses/complexes after weighting,^41^ software/hardware advancement,^42^ tracking assisted scanning,^43^ and machine learning automation,^44^ among others. Next, the OCT catheter and spatial coordinate catheter were passed through the same sheath and as such some movement or instability may have confounded the results. In addition, the current post-processing method that constructs the 3D models for CFD analysis requires tedious manual labor due to the enormous amount of images generated in each OCT acquisition. Computer vision and other machine learning techniques have been proposed for automatic tissue layer segmentation and could reduce the necessary manpower in the future.^29^^,^^45^^–^^47^

## 5 Conclusions

Given the lack of objective diagnostic tools and low rates of success in sleep surgery, a method to precisely and objectively determine sites of upper airway obstruction during sleep in OSA patients is warranted. This study demonstrated accurate anatomical structure, 3D remodeling, and calculations of airflow obstruction of a sleeping patient’s upper airway using OCT images. This OCT methodology provides valuable information regarding locations and degrees of upper airway obstruction during sleep, which can benefit patient decision-making and surgical outcomes in OSA. A comprehensive understanding of the physics of upper airway geometry will open new realms of research in diagnostic and therapeutic approaches to OSA that may rapidly transform the clinical management of these patients.

## Supplementary Material

10.1117/1.BIOS.1.3.035002.s01

10.1117/1.BIOS.1.3.035002.s1

10.1117/1.BIOS.1.3.035002.s2

## Data Availability

Code, data (including tabulated data), figures, and materials will be made available upon reasonable request.
