# Publisher’s Note: Optical coherence tomography to identify upper airway obstruction sites in an apneic patient

**DOI:** 10.1117/1.BIOS.1.3.039802

**Published:** 2024-12-17

**Authors:** Joseph C. Jing, Khodayar Goshtasbi, Yong Wang, Jason J. Chen, Erica Su, Ellen M. Hong, Katelyn D. Dilley, Yan Li, Frances B. Lazarow, Anthony Chin Loy, David Shamouelian, Said E. Elghobashi, Zhongping Chen, Brian J. F. Wong

**Affiliations:** a University of California Irvine, Beckman Laser Institute, Irvine, California, United States; b University of California Irvine, School of Biomedical Engineering, Irvine, California, United States; c University of California Irvine, Department of Otolaryngology-Head and Neck Surgery, Irvine, California, United States; d University of California Irvine, Department of Mechanical and Aerospace Engineering, Irvine, California, United States

## Abstract

The note clarifies a correction to the published article.

The original article was published in Volume 1 Issue 3 of *Biophotonics Discovery* (BIOS) on 28 November 2024 with some references listed in the supplemental material document that were not listed in the main reference list. To correct the oversight, those references were added as Refs. 48–53. Upon correction, the article was republished under the same DOI (10.1117/1.BIOS.1.3.035002) on 5 December 2024.

