# Supplementary material for: Optical coherence tomography to identify upper airway obstruction sites in an apneic patient
Source: Biophotonics Discov. 2024 Nov 28;1(3):035002. doi: 10.1117/1.BIOS.1.3.035002 (PMC13107249; doi:10.1117/1.BIOS.1.3.035002)
Supplement: Supplementary file 1 [file BIOS_001_035002_SD001.docx]

**Supplemental Material**

# *Detailed description of the direct numerical simulation – Lattice Boltzmann method (DNS-LBM)*

The main dependent variable of LBM is the fluid particle probability density distribution function, $f$, which is governed by the Boltzmann equation

$\frac{\partial f}{\partial t}+\xi\cdot\nabla f=\Omega$ (1)

where $t$ is time, $\xi$ is the velocity vector of the fluid particle, and $\Omega$ is the collision operation generally written in a differential-integral formulation even when only two-particle collision is considered.^1^

The macroscopic fluid properties, such as the fluid density, $\rho$, and the velocity, $u$, are functions of $f$ according to

$\rho=\int fd\xi, \rho u=\int f\xi d\xi$ (2)

The local pressure, $p$, is obtained from the equation of state for ideal gas where $R$ is the gas constant and $T$ is the absolute temperature.

$p=\rho RT$ (3)

In order to solve (1) numerically, $\Omega$ is simplified, and $f$ is discretized in the velocity space.

The flow in the complex geometry of the upper airway is 3D, time-dependent, undergoes transition from laminar to turbulent, and reverses its main direction with a respiratory cycle. In order to predict this flow accurately, we used direct DNS, which resolves all the relevant length- and time-scale and thus produces the most accurate details of turbulent flow properties. The conventional approach for DNS is DNS-NS, which solves the 3D Navier-Stokes (NS) equations numerically in simple geometries at moderate Reynolds numbers. However, in complex geometries such as that of the upper airway, it becomes computationally prohibitive for DNS-NS to resolve the flow in the near-wall regions. An alternative DNS approach is the DNS-LBM, which solves the discretized lattice Boltzmann equations and is well-suited for resolving all the relevant length- and time- scales of flows in complex geometries.^1,2^ The DNS-LBM has several advantages as compared to the conventional DNS-NS as will be discussed in the following section.

LBM has been introduced twenty years ago and developed rapidly in the past ten years. It has been used in simulating biomedical flows, such as flows in the respiratory system.^3–8^ In a previous publication, we studied the flows in two patient-specific upper airways, which were reconstructed from the CT scan data, via the DNS-LBM.^3^ Moreover, the time- averaged first spatial derivative of pressure (pressure gradient), $\frac{\partial p}{\partial}z$, was used to locate the region of the upper airway obstruction, while the time-averaged second spatial derivative, $\partial^{2}p/\partial z^{2}$, was used to pinpoint the exact location of the obstruction. Numerical results show that the DNS-LBM solver can be used to obtain accurate flow details in the upper airway and is a powerful tool to locate its obstruction.

In the BGK approach, the BGK collision operator, $\Omega_{BGK}$, is approximated as

$\Omega_{BGK}=-\frac{\left( f-f^{eq} \right)}{\tau'}$ (4)

where $f^{eq}$ is the local equilibrium distribution function, and $\tau'$ is the relaxation time. As such, (1) can be written via a finite set of velocity vector, $\mathbf{c}_{\alpha}$, as

$\frac{\partial f_{\alpha}}{\partial t}+\mathbf{c}_{\alpha}\cdot\nabla f_{\alpha}=\frac{f_{\alpha}^{\mathrm{eq}}-f_{\alpha}}{\tau'}$ (5)

where $\alpha$is the directional index of the discrete velocity vector $\mathbf{c}_{\alpha}$. The arrangement of $\mathbf{c}_{\alpha}$ in the velocity space is called a lattice, and in our 3D flow simulations the D3Q19 lattice is used, as shown in **Supplemental Figure 1**. $f_{\alpha}^{\mathrm{eq}}$ is a function of the local fluid density, velocity and sound speed, $c_{s}$, or temperature, and the formulation of $f_{\alpha}^{\mathrm{eq}}$ depends on the lattice structure.

Using the relation

$\frac{\partial x}{\partial t}=c$ (6)

the discretization of (5) in space and time gives

$f_{\alpha}\left( \mathbf{x}+\mathbf{c}_{\alpha}\delta x, t+\delta t \right)-f_{\alpha}\left( \mathbf{x},t \right)=\frac{f_{\alpha}^{\mathrm{eq}}(\mathbf{x},t)-f_{\alpha}(\mathbf{x},t)}{\tau}$ (7)

where $\mathbf{x}$ is the spatial coordinate of fluid particle, $\delta x$ and $\delta t$ are the lattice spacing and time increment, respectively, and $\tau=\tau^{'}/\delta t$ is the non-dimensional relaxation time, which is a function of the kinematic viscosity $\eta$_,_ $\delta t$, and $c_{s}$ according to

$\eta=c_{s}^{2}(\tau-0.5)\delta t$ (8)

The linear equation (7) is the governing equation of the BGK-LBM. The left-hand side of (5) describes the streaming motion of the fluid particles, and the right-hand side represents their collision. The integrals in (2) are then replaced by summations, and (3) is replaced by

$p=c_{s}^{2}\rho$ (9)

It should be noted that the incompressible NS equations can be obtained from (1), (5), and (7) using Chapman-Enskog expansion.^1^ In our simulation, the 3D computational domain is divided into a number of uniform 3D cells, which coincide with the lattice if $\delta x=\delta t=c=1$. The node at the center of each lattice is connected to the neighboring by $\mathbf{c}_{\alpha}$.

If the MRT collision operator, $\Omega_{MRT}$, is used instead of the $\Omega_{BGK}$the relaxation coefficient, $1/\tau$, on the right-hand side of (7) is replaced by a collision matrix, $\boldsymbol{\Lambda}$, such that

$f_{\alpha}\left( \mathbf{x}+\mathbf{c}_{\alpha}\delta x, t+\delta t \right)-f_{\alpha}\left( \mathbf{x},t \right)=\Lambda_{\alpha\beta}(f_{\beta}^{\mathrm{eq}}(\mathbf{x},t)-f_{\beta}(\mathbf{x},t))$ (10)

where

$\Lambda_{\alpha\beta}=\boldsymbol{\Lambda=}\mathbf{M}^{-1}\mathbf{SM}$ (11)

in which $\mathbf{M}$ is a given $m\times m$ transformation matrix for the DnQm lattice, $\mathbf{S}\boldsymbol{=}diag(s_{1},s_{2},\ldots,s_{m})$ and $s_{\alpha}$ can be determined by linear analysis and some physical parameters such as viscosity ^9^. $\Omega_{MRT}$ allows the solution of (10) to be more stable than that of (7) at the cost of slightly more computational time.

The linear LBM equations, (7) and (10), are simpler than the nonlinear NS equations and are readily parallelizable. As described by (3), moreover, the pressure is a local property, in the DNS-LBM, whereas in DNS-NS the pressure is obtained by solving the elliptic Poisson equation at a considerable computer cost.

In DNS-LBM, only the mesoscopic distribution functions, $f_{\alpha}$, need to be prescribed at the boundaries. An extrapolation method is adopted to treat straight or curved boundaries in the DNS-LBM solver.^10^ In this method, the distribution functions $f_{\alpha}$ are prescribed at the nodes adjacent to the boundary if that boundary intersects the planes connecting the lattice nodes. In particular, the distribution functions are decomposed into equilibrium and nonequilibrium parts. Fluid properties such as density, pressure and velocity at the external (outside the flow domain) nodes are determined through extrapolation of the internal fluid nodes nearest to the boundary and the physical boundary condition. The equilibrium parts of $f_{\alpha}$ are then calculated based on these properties. The nonequilibrium parts of $f_{\alpha}$ are approximated using an extrapolation based on the nonequilibrium parts at the neighbouring fluid nodes. Numerical tests show that such extrapolation method is of second order accuracy.^10^

The walls of the upper airway were assumed smooth and rigid, and the two-way interactions between the air flow and walls were not accounted for. The no-slip condition was imposed at the walls. Constant flow rates with uniform velocity profiles perpendicular to the inlet planes were prescribed at the inflow boundary. A pressure boundary condition, where the static pressure is assumed to be a constant value was applied at the outlet planes.

The computations were performed on Blue Waters (BW), a CrayXE6/XK7 supercomputer. A range of flow rates (2–50 L/min) was considered in the simulations. The 3D computational grid contained about 200 million nodes, and the solution required about 300,000 processor hours.

We have previously validated our DNS-LBM solver with both $\Omega_{BKG}$ and $\Omega_{MRT}$ by performing several simulations of canonical flows and obtained excellent agreements with the experimental or DNS data of these flows.^3^

**References**

1. Succi S. The lattice Boltzmann equation: for fluid dynamics and beyond. Oxford university press; 2001.

2. Sukop MC. DT Thorne, Jr. Lattice Boltzmann Modeling Lattice Boltzmann Modeling. 2006;

3. Wang Y, Elghobashi S. On locating the obstruction in the upper airway via numerical simulation. Respir Physiol Neurobiol. 2014;193:1–10.

4. Lintermann A, Meinke M, Schröder W. Investigations of human nasal cavity flows based on a Lattice-Boltzmann method. In: High Performance Computing on Vector Systems 2011. Springer; 2012. p. 143–58.

5. Eitel G, Freitas RK, Lintermann A, Meinke M, Schröder W. Numerical simulation of nasal cavity flow based on a Lattice-Boltzmann method. In: New Results in Numerical and Experimental Fluid Mechanics VII: Contributions to the 16th STAB/DGLR Symposium Aachen, Germany 2008. Springer; 2010. p. 513–20.

6. Hörschler I, Schröder W, Meinke M. On the assumption of steadiness of nasal cavity flow. J Biomech. 2010;43(6):1081–5.

7. Finck M, Hänel D, Wlokas I. Simulation of nasal flow by lattice Boltzmann methods. Comput Biol Med. 2007;37(6):739–49.

8. Ball CG, Uddin M, Pollard A. Mean flow structures inside the human upper airway. Flow, Turbul Combust. 2008;81:155–88.

9. D’Humières D, Ginzburg I, Krafczyk M, Lallemand P, Luo LS. Multiple-relaxation-time lattice Boltzmann models in three dimensions. Philos Trans R Soc A. 2002 Mar;360(1792):437–51.

10. Guo Z, Zheng C, Shi B. An extrapolation method for boundary conditions in lattice Boltzmann method. Phys fluids. 2002;14(6):2007–10.

**Supplemental Files**

**Supplemental Figure 1.** Schematic illustrations of a D3Q19 lattice.


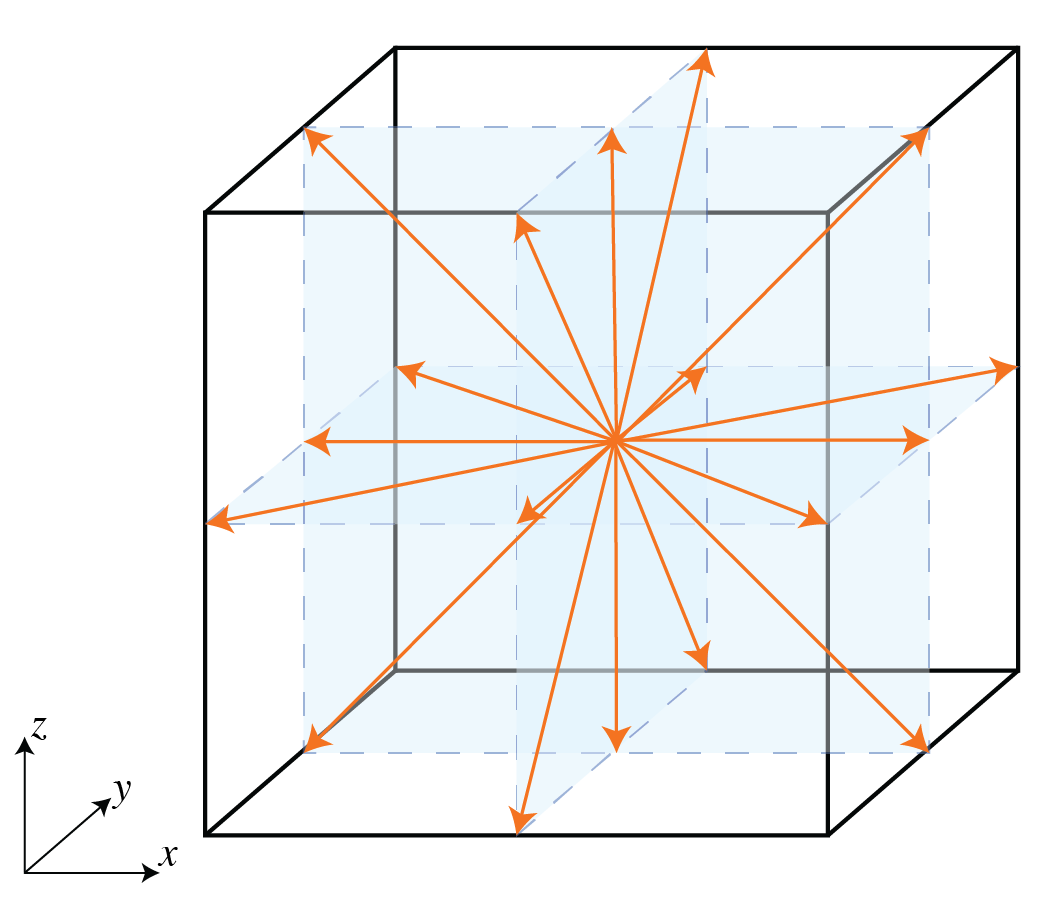


# **Supplemental Video 1.** Video of OCT pullback of the upper airway during wakefulness and sleep with annotations.

**Supplemental Video 2.** Video of flythrough of reconstructed 3D airway lumen with the corresponding OCT images.
